# Regulating Microstructure and Macroscopic Properties in Saturated Salt Solutions Containing Disordered Anions and Cations by Magnetic Field

**DOI:** 10.3390/molecules29020543

**Published:** 2024-01-22

**Authors:** Jihong Wang, Shasha An, Junchao Ren

**Affiliations:** 1 School of Physics and School of Materials Science and Engineering, East China University of Science and Technology, Shanghai 200237, China; 2 Department of Elementary Education, Hebei Normal University, Shijiazhuang 050024, China; 18303112890@163.com; 3 Shanghai Advanced Research Institute, Chinese Academy of Sciences, Shanghai 201210, China

**Keywords:** weak static magnetic fields, excess Raman spectroscopy, contact angle, X-ray diffraction, molecular dynamics simulations

## Abstract

Saturated aqueous salt solutions have diverse applications in food production, mineral processing, pharmaceuticals, and environmental monitoring. However, the random and disordered arrangement of ions in these solutions poses limitations across different fields. In this study, we employ magnetic fields to regulate the disordered arrangement by a comprehensive methodology combining contact angle measurement, Raman spectroscopy, X-ray diffraction, and molecular dynamics simulations on saturated KCl solutions. Our findings reveal that weak magnetic fields impede the formation of K-Cl contact pairs and disrupt hydrogen bond networks, particularly DDAA and free OH types. However, they facilitate the interaction between water molecules and ions, leading to an increase in the number of K-O and Cl-H contact pairs, along with an expansion in ion hydration radius. These changes affect macroscopic properties, including the interaction with solid substrates and potential solubility increases. Our experimental and simulation results mutually validate each other, contributing to a theoretical framework for studying magnetic field–material interactions.

## 1. Introduction

Saturated salt solutions are crucial for various chemical and biological processes, including food production, cosmetics manufacturing, energy utilization, pharmaceuticals, and environmental monitoring [1,2,3,4]. However, the random and disordered arrangement of ions in these solutions poses limitations across different fields. For instance, in chemical synthesis, the disorderly ion arrangement can destabilize crystal morphology and size, thereby impacting product purity and properties [5]. Disordered salt solutions also restrict conductivity in electrolytes and batteries, limiting the energy density and life cycle [6]. Additionally, in drug delivery and biomedical applications, disordered salt solutions can decrease drug stability and bioavailability, affecting therapeutic efficacy [7]. Regulating ion arrangements in saturated salt solutions enables the transformation from disorder to order, overcoming these challenges and expanding potential applications. Magnetic fields offer the benefits of convenience, energy efficiency, and environmental friendliness. Therefore, employing magnetic fields to regulate disordered ions in saturated salt solutions holds significant promise for diverse applications.

Research on the influence of magnetic fields on conventional inorganic salt aqueous solutions has made progress [8,9,10,11]. However, studies exploring the effects of magnetic fields on the structure and properties of solutions have mainly solely relied on either experimental methods [12,13,14,15,16,17] or molecular dynamics (MD) simulations [18,19,20,21]. Moreover, there is a lack of research on the microstructures of saturated electrolyte solutions containing disordered anions and cations under weak magnetic fields, particularly when considering combined experimental and simulation approaches. Therefore, it calls for the study of regulating the microstructure and macroscopic properties of saturated salt aqueous solutions containing disordered anions and cations by weak magnetic fields. The effects of a magnetic field on the microstructures of KCl aqueous solutions with varying concentrations were investigated by the current authors in a previous study [22]. The results indicated that at very low mass fractions of KCl aqueous solutions, the magnetic field primarily affects hydrogen bonding. However, as the mass fraction increases, the magnetic field predominantly enhances the interaction between ions and water molecules.

Here, we extend our study on how the microstructure and macroscopic properties of salt solutions containing disordered ions are regulated by weaker magnetic fields at saturation. To accomplish this, we introduced magnetic field strengths of 0.1 T, 0.2 T, and 0.3 T. In this work, we adopted a combined approach utilizing experiments (contact angle determination, Raman spectroscopy, and X-ray diffraction (XRD)) and MD simulations to comprehensively examine the regulating effects on the macroscopic properties and microstructure of saturated potassium chloride (KCl) aqueous solutions by weak magnetic fields. The results bridge the knowledge gap regarding the physical properties and microstructural changes of saturated potassium chloride aqueous solutions under weak magnetic fields. Additionally, this integrated experimental and computational methodology provides a methodological and technical framework for investigating the effects of magnetic fields on other substances. Moreover, the findings can offer valuable guidance for experimental design and data analysis in similar research domains, expanding the potential for manipulating material properties using magnetic fields.

## 2. Results and Discussion

### 2.1. Raman Spectroscopy Results and Analysis

**Figure 1:**
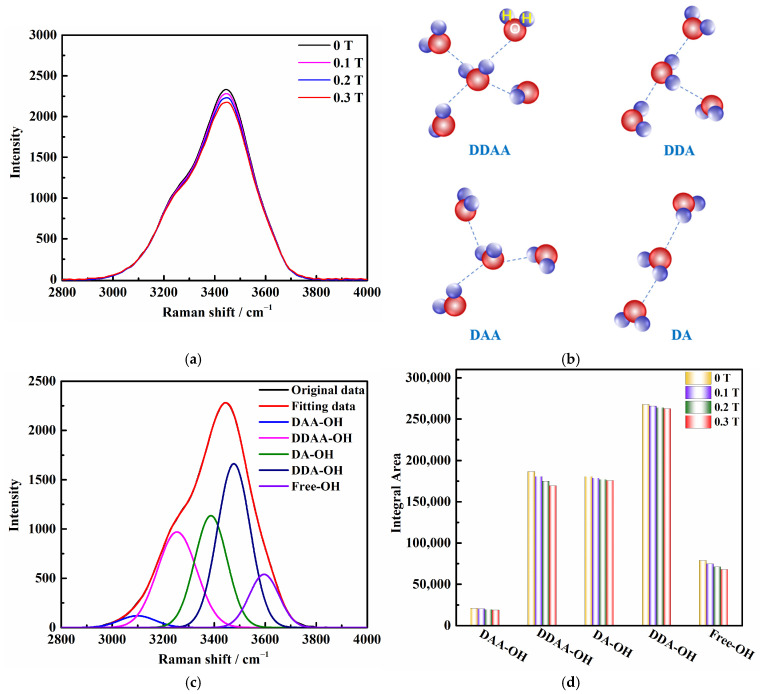
(**a**) Raman spectroscopy results of saturated KCl aqueous solution under 0 T, 0.1 T, 0.2 T, and 0.3 T. (**b**) O–H stretching band structures in aqueous solution: DDAA, DDA, DAA, and DA. (**c**) Five individual Raman positions obtained from Gaussian functions representing five O–H stretching bands of saturated KCl aqueous solution under 0 T. (**d**) Raman peak area changes of saturated KCl aqueous solution under 0 T, 0.1 T, 0.2 T, and 0.3 T at room temperature.

To analyze the O-H stretching vibration pattern in Raman spectroscopy at room temperature, we focused on the spectral region of 2800 cm^−1^ to 4000 cm^−1^. Figure 1a illustrates that the Raman strength (*Y*-axis) of the magnetized solution decreased compared to the unmagnetized saturated KCl aqueous solution, while the frequency (*X*-axis) remained unchanged. To understand the differences in the behavior of O-H stretching in Raman spectroscopy, we employed a deconvolution process using Gaussian distribution. The Raman OH stretching band was deconvoluted into five sub-bands at 3051, 3255, 3385, 3477, and 3595 cm^−1^ in Figure 1c, which can be assigned to 𝑣_DAA–OH_, 𝑣_DDAA–OH_, 𝑣_DA–OH_, 𝑣_DDA–OH_, and free OH symmetric stretching vibrations, respectively (see Figure 1b) [23]. Under ambient conditions, the main local hydrogen bonding motifs for a water molecule can be classified as DDAA (double donor–double acceptor), DDA (double donor–single acceptor), DAA (single donor–double acceptor), and DA (single donor–single acceptor). The change in magnetic field intensity resulted in corresponding variations in the peak areas (Figure 1d). With an increase in the magnetic field, the proportion of each hydrogen bond structure area decreased, indicating a gradual decrease in each type of hydrogen bond structure under the influence of the magnetic field, particularly the DDAA and free OH types of hydrogen bond structures.

**Figure 2:**
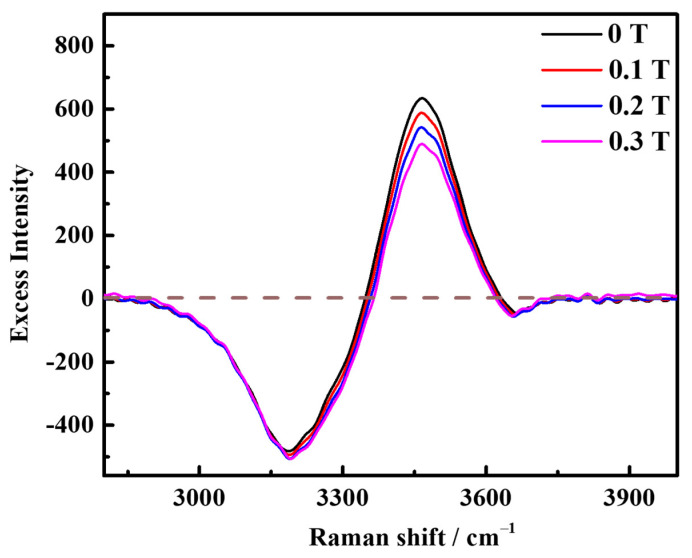
Excess Raman spectroscopy of saturated KCl aqueous solution under 0 T, 0.1 T, 0.2 T, and 0.3 T at room temperature.

The excess Raman spectroscopy [24,25] was obtained by subtracting the Raman spectroscopy of water, as shown in Figure 2. This excess Raman spectroscopy displays both positive and negative peaks, with the negative peaks indicating the structural damage of the aqueous solution [26]. Specifically, negative peaks were observed at 3195 cm^−1^ and 3650 cm^−1^, suggesting that the addition of saturated concentration KCl resulted in the disruption of the original DDAA type and free OH type hydrogen bond structures in water. Furthermore, the increase in peak area indicated that the damage became more severe with the increase in the magnetic field, which aligns with the results shown in Figure 1. Additionally, the positive peaks in the excess Raman spectroscopy signify the presence of ion pairs in the solution [24]. A positive peak appeared at 3465 cm^−1^ in the excess Raman spectroscopy and the peak area decreased, indicating that the magnetic field reduced the formation of contact pairs between K-Cl ions in the original solution. Moreover, the slight shift in peak position indicates that the addition of the magnetic field alters the relative content of indirect and direct contact ion pairs in the solution [26,27].

### 2.2. Contact Angle Results Analysis

**Figure 3:**
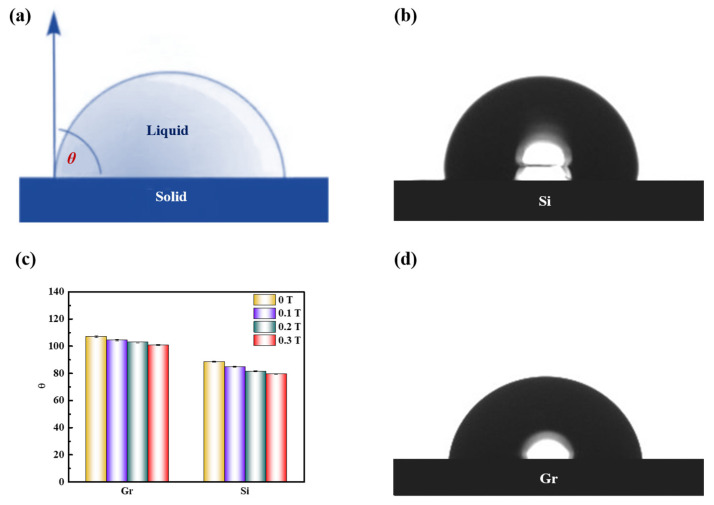
(**a**) Schematic diagram of the contact angle; (**c**) Contact angle variation diagram of saturated KCl aqueous solution under 0 T, 0.1 T, 0.2 T, and 0.3 T at room temperature on graphite (Gr) and silicon (Si) surface; (**b**,**d**) Contact angle diagram of saturated KCl aqueous solution on silicon and graphene surface at 0 T.

The application of a magnetic field induces changes in the distribution of water molecules and the clustering state within a solution, which inevitably impacts its macroscopic properties, including surface tension. To investigate the alterations in molecular aggregation and surface tension, we measured the contact angle (Figure 3a) of a saturated KCl aqueous solution on different solid surfaces (graphite and silicon) during magnetic treatment (Figure 3b–d). Interestingly, the contact angle of the magnetized saturated KCl solution was slightly smaller than that of the unmagnetized solution on both graphite and silicon surfaces. Although the difference in macroscopic characteristics appears small, it surpassed the accuracy range of the instrument, indicating that the discrepancy cannot be attributed to instrumental error alone. This suggests that after exposure to a static magnetic field, the saturated potassium chloride aqueous solution exhibits enhanced infiltration onto the surfaces of graphite and silicon materials. Consequently, stronger adhesion between the liquid and solid surface promotes the greater expansion of the liquid droplet on the solid surface, resulting in smaller surface tension. Notably, surface tension is closely linked to the nature of matter; the surface tension of a liquid is an outcome of intermolecular interactions. Hence, the introduction of a weak static magnetic field appears to reduce the intermolecular forces within the saturated KCl aqueous solution.

### 2.3. Analysis of XRD Results

**Figure 4:**
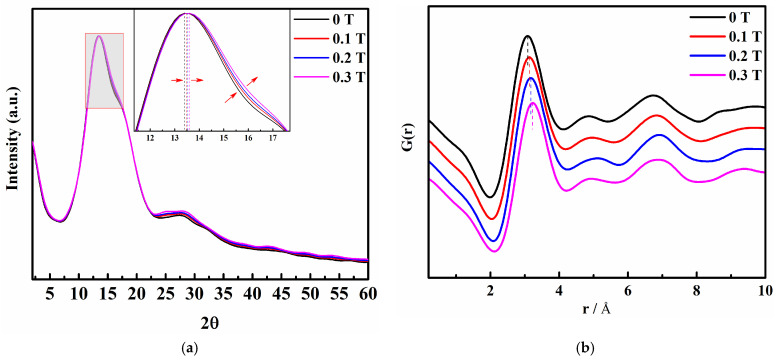
(**a**) X-ray diffraction (XRD) pattern of a saturated KCl aqueous solution under 0 T, 0.1 T, 0.2 T, and 0.3 T; (**b**) Reduction pair distribution function $G\left(r\right)$ profiles of saturated KCl aqueous solution under 0 T, 0.1 T, 0.2 T, and 0.3 T.

According to Figure 4a, the addition of a magnetic field to the saturated KCl aqueous solution results in a blue shift between 10° and 19° in the XRD pattern, accompanied by a gradual increase in the half-peak width. Additionally, there is an increase in peak intensity between 22° and 35° as the magnetic field strength increases. These observations suggest that the internal structure of the saturated KCl aqueous solution undergoes slight changes under the influence of the static magnetic field. To confirm this, we proceeded to perform the RDF analysis of the XRD data.

The $G\left(r\right)$ profiles (Figure 4b) of the saturated KCl aqueous solution under 0 T, 0.1 T, 0.2 T, and 0.3 T were calculated from the XRD intensity data. It is evident that the addition of a magnetic field gradually decreases the peak value at 1.5 Å in the XRD pattern. Simultaneously, the peaks ranging from 2.15 Å to 4.25 Å shift towards higher values of r, indicating that weak static magnetic fields alter the interatomic distances in the system. This range encompasses multiple atomic distance information. By changing the atomic distances, it is inferred that the magnetic field influences the microstructure of the saturated KCl aqueous solution. Moreover, an increase in magnetic field intensity accelerates the disruption of existing ion contact pairs and the hydrogen bond network structure, while promoting the formation of new contact pairs. To investigate the creation or destruction of ion pairs in greater detail, we employed MD simulations to analyze and interpret the results.

### 2.4. Simulation Results Analysis

**Figure 5:**
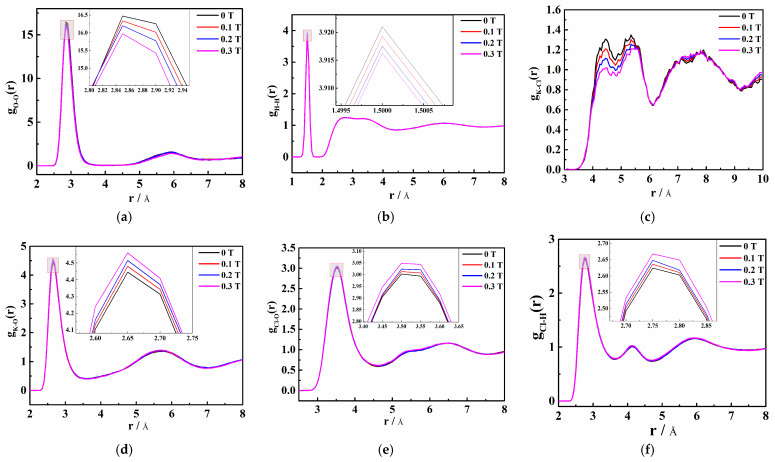
Radial distribution function (RDF) of each ion distance in the saturated KCl aqueous solution under 0 T, 0.1 T, 0.2 T, and 0.3 T and their local magnifications, namely (**a**) O-O, (**b**) H-H, (**c**) K-Cl, (**d**) K-O, (**e**) Cl-O, and (**f**) Cl-H.

As can be seen in Figure 5a, the first peak of *g*_O-O_(*r*) is approximately 2.85 Å. With the increase in magnetic field, the peak weakens and the peak area decreases, which makes the coordination numbers of water molecules in the saturated KCl aqueous solution decrease. Figure 5b shows that the first peak of *g*_H-H_(*r*) is at 1.50 Å and decreases with increasing magnetic field. This indicates that magnetic fields cause the hydrogen bond interaction in the saturated KCl aqueous solution to weaken, which is contrary to the general belief that magnetic fields can enhance the hydrogen bonding effect in pure water or low-concentration electrolyte solutions [28].

The first and second peaks of *g*_K-O_(*r*) in Figure 5d are approximately 2.65 Å and 5.65 Å, respectively. It reveals that the division of the first and second hydration cycles of K^+^ is obvious. In addition, the first hydration circle is 2.65 Å away from K^+^ and the oxygen atom is close to K^+^. As the peak intensity increases, it can be deduced that with the increase in the magnetic field, the number of oxygen atoms around K^+^ increases, making the combination of K^+^ and oxygen atoms tighter. The first and second peaks of *g*_Cl-O_(*r*) in Figure 5e are around 3.55 A and 5.35 A, respectively, while the first and second peaks of *g*_Cl-H_(*r*) in Figure 5f appear at approximately 2.75 Å and 4.15 Å, respectively. This means that there are at least two hydration layers around Cl^−^, too. Moreover, the first hydration layer is 2.75 Å away from Cl^−^ and the hydrogen atom is close to Cl^−^. The second hydration layer is still approached by the hydrogen atoms to the Cl^−^. The larger the magnetic field, the more hydrogen atoms around Cl^−^, indicating that the addition of the magnetic field promotes the interaction between Cl^−^ and hydrogen atoms. Compared with *g*_Cl-H_(*r*), the first peak of *g*_K-O_(*r*) has a larger peak intensity, a smaller valley value, and a narrower peak, which indicates that the hydration ability of K^+^ is greater than that of Cl^−^. The solute–solvent RDFs reveal that weak static magnetic fields promote the interaction between ions and water molecules in saturated aqueous solutions of inorganic salts. Therefore, the number of water molecules in the ion hydration layer increases and the ion hydration radius increases.

**Figure 6:**
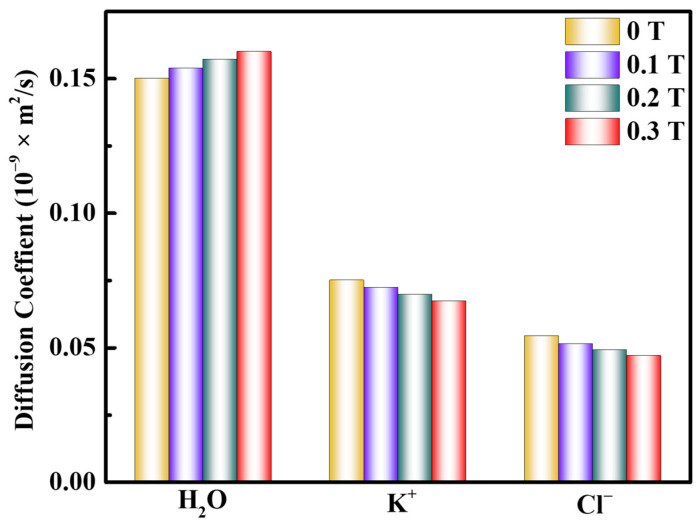
Diffusion coefficient of H_2_O, K^+^, and Cl^−^ in the saturated KCl aqueous solution under 0 T, 0.1 T, 0.2 T, and 0.3 T.

Figure 5c shows that the first peak of *g*_K-Cl_(*r*) in the saturated KCl aqueous solution is 4.45 Å. It is larger than that of *g*_K-O_(*r*) and *g*_Cl-H_(*r*). This result indicates that if Cl^−^ is regarded as the central ion, there will be K^+^ outside its first hydration layer and water molecules between K^+^ and Cl^−^. Magnetization results in a decrease in the number of interacting K-Cl ion pairs in the saturated KCl aqueous solution. This implies that the probability of ions appearing around ions is reduced, making the interaction between ions and water molecules stronger. Moreover, the self-diffusion coefficient of water molecules increases as the magnetic field increases, while that of K^+^ and Cl^−^ decrease (Figure 6). It confirms that the magnetic influence facilitates the disruption of hydrogen bonds by ions, leading to enhanced ion-water molecule interactions, thereby reducing the number of hydrogen bonds and increasing ion hydration radius in the saturated KCl aqueous solution.

The response of aqueous electrolyte solutions to magnetic fields can be categorized into two aspects: the impact on hydrogen bonding between water molecules influenced by the magnetic field and the response of ions to the magnetic field [29,30,31]. The influence of the magnetic field on the microstructure of saturated KCl aqueous solution is mainly manifested in hydrogen bonding, interionic interaction, and interaction between ions and water molecules. Both K^+^ and Cl^−^ possess a structural disorder, resulting in weak interactions with water molecules and limited impact on the hydrogen bond network of water in the absence of a magnetic field. However, the addition of a magnetic field induces a more pronounced response from K^+^ compared to Cl^−^. The heightened magnetic field expedites the motion of both K^+^ and Cl^−^, resulting in extensive disruption of the hydrogen bond network structure of adjacent water molecules as well as the contact pair between K^+^ and Cl^−^. Consequently, the interaction between ions and water molecules within the saturated KCl aqueous solution becomes more pronounced and stronger.

## 3. Materials and Methods

### 3.1. Magnetization

The experiment utilized a standard KCl reagent of exceptional purity (>99.99%), manufactured by the Macklin Company, Shanghai, China. Ultrapure water was obtained by our lab’s ultrapure water machine (ULUPURE), exhibiting an impressive electrical resistivity of 18.25 MΩ/cm. To obtain a saturated solution, a beaker was initially filled with 100 mL of ultrapure water and stirred continuously at room temperature. Subsequently, KCl was incrementally added in small portions multiple times until no further dissolution occurred. The quantity of KCl added was documented. This protocol was repeated three times and the average mass of KCl was determined.

**Figure 7:**
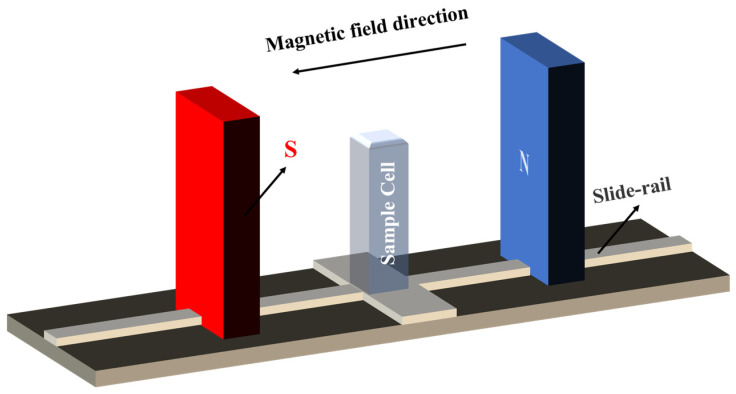
Schematic diagram of the experimental setup.

The alterations in the physical properties of magnetized saturated KCl aqueous solutions were investigated through variations in the strength of the magnetic field. A pair of neodymium magnets (50 × 105 × 30 mm) was utilized to generate a magnetic field and the intensity of the field was adjusted by manipulating the position of the magnets on the slide rail (see Figure 7). The proximity of the two magnets enabled the generation of uniform magnetic fields of 0.1 T, 0.2 T, and 0.3 T and the precise positions of these fields were determined using the HT20 digital Tesla instrument, manufactured by Shanghai Hengtong Magnetoelectric Technology Co., Ltd., Shanghai, China. To achieve a thorough magnetization, a quartz colorimetric dish (dimensions: 45 × 12.5 × 12.5 mm^3^; volume: 3.500 mL; light path: 10 mm) filled with the saturated KCl aqueous solution was placed at these positions for 3 h. All samples were sealed while being magnetized under room temperature conditions. The density of the solution was determined via the weighing method, with the average value of three trials being recorded. The density of the saturated KCl aqueous solution was measured to be 1.1798 g/cm^3^. Following the magnetization process, we conducted a comprehensive investigation into the physical properties and microstructure of the magnetized saturated KCl aqueous solution, comparing it with the unmagnetized solution (0 T). For this purpose, this study employs a comprehensive approach integrating experimental methodologies for measuring contact angles, Raman spectroscopy, and XRD, together with theoretical methods of MD simulations.

### 3.2. Raman Spectroscopy Experiment

In this study, Raman spectroscopy was employed to investigate the changes in the molecular structure of saturated KCl aqueous solutions under various magnetic field conditions. Raman spectroscopy is a well-established technique used to probe the molecular structure of materials. The experiments were carried out at room temperature using a laser Raman spectrometer (manufactured by Brock AG, Ettlingen, Germany). The excitation wavelength used was 532 nm, with an operating power of 10 mW. Raman spectroscopy was collected by performing 64 scans at a resolution of 1 cm^−1^, utilizing a 50× objective on the microscope. Then, the normal Raman spectroscopy of saturated KCl aqueous solutions magnetized with different static magnetic fields at room temperature was measured.

### 3.3. Contact Angle Measurement

When the liquid is unable to spread on a solid surface, it forms a specific contact angle at the interface of the liquid, solid, and gas phases. Utilizing a camera system, we obtained contour images of the liquid on the solid surface, which were then processed using image processing technology and various calculation methods to calculate the contact angle between the liquid and the solid. The static contact angle was measured by the sessile drop method, where a droplet was dispensed from a capillary tube onto the upper surface of the solid and the angle formed by the droplet on the solid surface was collected and analyzed. We conducted contact angle measurements using the OCA40 micro-optical video contact angle measuring instrument. A saturated KCl aqueous solution was applied to different solid surfaces after subjecting them to magnetic fields of 0 T, 0.1 T, 0.2 T, and 0.3 T for a duration of 3 h. The injected samples had a volume of 0.5 µL. Contact angles were measured within the range of 0 to 180°. Two types of interfaces, silicon (Si) and graphite (Gr), were selected to measure the contact angles at five different locations on the solid surfaces and the results were averaged.

### 3.4. XRD Measurement and Data Analysis Utilizing PDFgetX3 Software

XRD measurements were conducted at the Shanghai Synchrotron Radiation Facility (SSRF) using the Huber 5021 six-circle diffractometer in BL14B (Shanghai, China). An 18 KeV X-ray beam, corresponding to a wavelength λ of 0.6889 Å, was generated by a sagittal double crystal monochromator Si (111). The device exhibited an energy resolution of up to 2 × 10^−4^@10 KeV. To precisely quantify the scattered radiation, we employed the CsI scintillation crystal detector. The magnetized samples were carefully positioned within a capillary (Charles Super Company, Westborough, MA, USA) featuring a diameter of 0.01 mm and wall thickness of 2.0 mm, ensuring precise sample alignment. These capillaries were then securely placed onto the goniometer head holder for further measurement. Subsequent adjustments were made in both the vertical and horizontal directions using the goniometer, effectively centering the sample within the diffraction circle to guarantee the reliability of the collected scattering measurement data. During the scan, a 0.2° increment and a scan time of 1 s/step were employed, with Slit S5 fully opened, and a spot size of 0.3 mm at a controlled temperature of 25 °C. To ensure measurement accuracy, three parallel scans were performed and the resulting average was taken as the final measurement.

The XRD intensity data comprise coherent, incoherent, multiple, and background scattering contributions but only coherent scattering possesses the structural information of the solution. By utilizing the PDFgetX3 version 2.1.1 [32,33], the structural information of the solution was extracted by subtracting the incoherent, multiple, and background scattering data from the overall scattering intensity. This process ultimately yielded the total scattering structure function $S\left(Q\right)$. The Fourier transformation was subsequently conducted on the resulting differential structure function $F\left(Q\right)$ ($F\left(Q\right)=Q\left[F\left(Q\right)-1\right]$), leading to the determination of the reduced pair distribution function $G\left(r\right)$. Finally, the $G\left(r\right)$ [33] function was acquired by

(1) $$G\left(r\right)=4\pi r\rho_{0}\left(g\left(r\right)-1\right)=\frac{2}{\pi }\int_{0}^{\infty }Q\left[S\left(Q\right)-1\right]\sin\left(Qr\right)dQ$$

where $G\left(r\right)$ represents the atom pair distribution function (PDF), r is the distance between two atoms, $\rho_{0}$ denotes the number density of atoms in this system, and Q is the diffraction vector or momentum transfer.

### 3.5. MD Simulations and Data Analysis

MD simulations were conducted to investigate the behavior of saturated aqueous KCl solutions at 25 °C under magnetic fields of 0 T, 0.1 T, 0.2 T, and 0.3 T. Initially, a water box was created using a construction function by Materials Studio version 8.0 (MS) software based on the molar ratio of KCl to water molecules (46:529). It consists of 46 ion pairs alongside 529 water molecules, with the arrangement of K^+^ and Cl^−^ ions in proximity to the water molecules being randomized. The simulation box was constructed as a cube with a side length of 26.3195 Å, corresponding to a density of 1.1798 g/cm^3^. The COMPASS II force field was employed. The simulated temperature was 298 K and the thermostat was the Nose–Hoover method. The NVT ensemble was selected and the electrostatic force and van der Waals force were calculated using the Ewald method and the atom-based method, respectively. Each atom was assigned an initial random velocity. Additionally, a multistage temporal simulation approach was utilized [34]. In the initial phase, the system was allowed to equilibrate for 1000 ps without any applied magnetic field, with a time step of 0.1 fs. Subsequently, the magnetic fields of 0 T, 0.1 T, 0.2 T, or 0.3 T were applied and the simulations were conducted for 5000 ps with a time step of 0.1 fs. The applied magnetic field was oriented along the *Z*-axis and influenced the movement of molecules within the X-Y plane. The system reached equilibrium, where the energy and characteristics remained constant until the end of the simulation. Finally, the radial distribution function (RDF) was calculated based on the equilibrium model. RDF [35] is a valuable tool used to extract detailed atomic-level structural information from MD simulations. Specifically, it allows for the identification of the first coordination shell surrounding the central atom, which is characterized by the first peak observed in the RDF plot. The coordination number [36], which refers to the count of atoms within the first coordination layer, can be achieved by multiplying the area underneath the first peak by the average density. In the RDF, the symbol “r” corresponds to the interatomic distance between atoms, denoted as “atom-atom”. Examples of such interatomic distances include O-O, H-H, K-O, Cl-O, Cl-H, and K-Cl.

Moreover, the ion self-diffusion coefficient is acquired by the Einstein method [37], which can be expressed as

(2) $$D=\underset{t\to \infty }{\lim}\frac{1}{6Nt}\langle \sum_{i=1}^{N}{Ⅰr_{i}(t)-r_{i}(0)Ⅰ}^{2}\rangle$$

where N is the number of atoms diffused in the system, t is for time, $r_{i}(t)$ represents the instantaneous displacement, and $r_{i}(0)$ represents the original displacement. The fundamental principle here is to solve the differential equation of ion motion by the numerical method. Subsequently, the curve depicting the mean square displacement (MSD) to time is obtained through statistical averaging. Finally, the self-diffusion coefficient (D) is determined by calculating one-sixth of the slope (a) of this curve. Equation (2) can be simplified as

(3) $$D=\frac{a}{6}$$

where a is the slope of the MSD to time curve obtained by MD simulation and D is the self-diffusion coefficient. Distinguishing between ballistic and diffusive regimes is possible by plotting MSD versus time on a log–log scale. When the MSD increases linearly with time, it indicates that ions are undergoing free diffusion, where no significant restrictions hinder their movement. Conversely, the non-linear growth of MSD suggests the presence of limitations or constraints in the system. On the other hand, it is a common practice to use more than one time origin to obtain better accuracy in the determination of the MSD curves. Choosing the right time interval is also crucial for obtaining accurate MSD results.

## 4. Conclusions

In summary, we employed weak magnetic fields to regulate the disordered arrangement in saturated KCl solutions through a comprehensive methodology involving contact angle measurement, Raman spectroscopy, XRD, and MD simulations. It can be found that even a weak magnetic field can induce a transition of ions from a disordered state to an ordered state. The increase in magnetic field intensity reduces the number of contact pairs between K and Cl within the solution, while also causing more damage to the various hydrogen bond structures, specifically the DDAA and free OH types. However, it enhances the interaction between water molecules and ions, resulting in more K-O and Cl-H contact pairs. Changes in the microstructure can lead to modifications in macroscopic properties. The presence of a magnetic field strengthens the interaction force between a solid substrate and a saturated aqueous solution of KCl. This discovery may enhance our theoretical understanding of the effects of weak magnetic fields on the physical properties of electrolyte solutions containing disorderly anions and cations, such as conductivity, solubility, and evaporation. This multifaceted approach, combining experimental investigations with computational techniques, provides a robust framework for exploring the intricate interplay between magnetic fields and various materials. Additionally, it offers valuable guidance for experimental design and meticulous data analysis. This research amplifies the potential of utilizing magnetic fields to manipulate material properties across diverse fields such as chemical production, separation and purification processes, geological exploration, and the highly intricate domain of pharmaceutical research and development.

## Data Availability

The data presented in this study are available in the article.
